# The onchocerciasis hypothesis of nodding syndrome

**DOI:** 10.1371/journal.pntd.0011523

**Published:** 2023-08-17

**Authors:** Robert Colebunders, Amber Hadermann, Joseph Nelson Siewe Fodjo

**Affiliations:** Global Health Institute University of Antwerp, Antwerp, Belgium; IRCCS Sacro Cuore Don Calabria Hospital, ITALY

## Abstract

Nodding syndrome (NS) is a phenotypic presentation of onchocerciasis-associated epilepsy (OAE). OAE is an important public health problem in areas with high ongoing *Onchocerca volvulus* transmission. OAE, including NS, is preventable by strengthening onchocerciasis elimination programs. The presence of tau in OAE postmortem brains could be the consequence of neuroinflammation directly or indirectly induced by *O*. *volvulus*. Omics research is needed to investigate whether *O*. *volvulus* worms contain a neurotropic virus.

## Introduction

Nodding syndrome (NS) was initially believed to be a unique condition that was restricted to certain areas in Tanzania, northern Uganda, and South Sudan and thought to be linked to certain events or living conditions in those areas (e.g., war and displacement of persons in camps) [1].

In recent years, NS cases have been reported in many other countries, all of which had a high level of ongoing or past *Onchocerca volvulus* transmission [2]. To determine the cause of NS, it is important to investigate whether this condition could be part of a wider clinical spectrum. The latter is indeed suggested by a growing number of epidemiological studies and the following arguments [2].

1. NS and Nakalanga syndrome (characterized by morphological deformities, retarded growth, and delayed/absent secondary sexual development) appear in the same onchocerciasis-endemic areas with high *O*. *volvulus* transmission, together with high numbers of other forms of epilepsy with similar characteristics except head nodding seizures [2]. These characteristics are the criteria of the onchocerciasis-associated epilepsy (OAE) case definition proposed for epidemiological studies. This form of epilepsy appears in previously healthy children between the ages of 3 and 18 years, without an obvious cause for epilepsy, in an onchocerciasis-endemic region with high ongoing *O*. *volvulus* transmission [2]. Only a relatively small proportion of individuals with OAE present with NS, the most debilitating form of OAE associated with most severe cognitive impairment [3].
2. The NS epidemic in northern Uganda [4] and South Sudan [5] appeared together with an epidemic of other forms of epilepsy meeting criteria of OAE.
3. Nodding and Nakalanga syndromes are often observed in families with siblings with other forms of OAE and may be associated with blindness [6].
4. Both NS and other forms of OAE present with similar cerebral and cerebellar atrophy on magnetic resonance imaging [7]. Persons with NS may have a higher degree of global cerebral atrophy, but this may be related to a longer duration of epilepsy [7]. In postmortem studies, NS and OAE also present with similar pathological findings [8].

## Findings suggesting that *O*. *volvulus* directly or indirectly may induce epilepsy

1. A case control study in the Mbam valley, an onchocerciasis-endemic region in Cameroon, revealed more intense infections with *O*. *volvulus* in persons with epilepsy than in nonepileptic controls and a strong positive association between community microfilarial (mf) load and epilepsy prevalence. In addition, the study also found an inverse relationship between villages’ distance from the river (breeding site for the blackfly vectors) and epilepsy prevalence [9]. Also, in South Sudan, the highest epilepsy prevalence was observed among households living close to blackfly breeding sites, and families at these sites often had several children with OAE [5,6].
2. In population-based surveys in onchocerciasis-endemic areas, a positive association between *O*. *volvulus* prevalence and the prevalence of epilepsy was observed [10]. A meta-analysis of 8 population-based studies in onchocerciasis-endemic areas, conducted before 2008, showed that the epilepsy prevalence increased, on average, by 0.4% for each 10% increase in onchocerciasis prevalence [10].
3. In 2 cohort studies in Cameroon, a temporal and mf dose-dependent association was observed between the level of *O*. *volvulus* infection in early childhood and the development of epilepsy later in life [11].
4. Microfilaria have been observed in the cerebrospinal fluid (CSF) of persons with *O*. *volvulus* infection in studies conducted prior to introduction of community drug treatment with ivermectin (CDTi) [12].
5. Epidemics of epilepsy emerged in onchocerciasis-endemic areas with no or little ivermectin distribution or where the onchocerciasis elimination programme had been interrupted [4–6].
6. Successful onchocerciasis elimination strategies reduced the incidence of epilepsy including NS in onchocerciasis-endemic regions, as was observed in northern Uganda Mahenge and Maridi, and in western Uganda. OAE stopped appearing once onchocerciasis was eliminated (Table 1).

**Table 1:** Sites where onchocerciasis interventions were associated with reduced epilepsy/NS burden.

			Preintervention incidence		Postintervention incidence	
Study site	Intervention	Survey periods	Epilepsy cases* per 100,000 person-year	NS cases per 100,000 person-year	Epilepsy cases* per 100,000 person-year	NS cases per 100,000 person-year
Northern Uganda [4]	Annual CDTi + vector control	2012–2017	1,165 (95% CI: 621–2,117)	490 (95% CI: 176–1,232)	130 (95% CI: 15–630)	43 (95% CI: 0–490)
Western Uganda [13]	CDTI + vector control	1994–2018	418 (95% CI: 265–626)	Not documented	73 (95% CI: 32–114)	NS stopped to appear
Mahenge, Tanzania [14]	Biannual CDTi high coverage	2017/2018–2021	177.6 (121.2–258.5)	18.4 (4.7–58.5)	45.5 (22.2–89.7)	177.6 (121.2–258.5)
Maridi, South Sudan [15]	Biannual CDTi low coverage + “Slash and Clear” vector control	2018–2022	348.8 (95%CI: 307.2–395·8)	154.7 (95% CI: 127.6–187.3)	41.7 (95% CI: 22.6–75.0)	10·4 (95% CI: 2.7–33.2)

CDTi, community directed treatment with ivermectin; NS, nodding syndrome.

*All epilepsy cases, including NS.

## Pathogenesis of OAE

While there is a very strong epidemiological association between onchocerciasis and epilepsy, the exact pathophysiology of OAE, including NS, is still unknown. A plausible explanation for the OAE pathology is that the epilepsy is induced by *O*. *volvulus* mf occasionally penetrating the brain of heavily infected young children. Indeed, before CDTi was implemented, mf were detected in CSF, e.g., in 1976 by Duke in Cameroon in persons with high *O*. *volvulus* [12] mf loads. It is unlikely that the CSF was contaminated with mf from the skin in this study, because the first 5 to 6 drops of CSF were discarded [12]. Additionally, the intensity of mf infection in the CSF increased from 2 mf/ml to 19 mf/ml after administration of diethylcarbamazine (DEC) [12]. Six persons with a high concentration of mf in CSF (8 to 31 mf/ml) developed severe vertigo and one of them a temporary parkinsonian condition. DEC is known to cause inflammation, which could increase blood–brain barrier (BBB) permeability [16]. This increased permeability might make it easier for mf to penetrate the central nervous system (CNS). Duke hypothesized that mf enter the CSF through the capillary wall of the choroid plexus in the lateral, third, and fourth ventricles [12].

In more recent postmortem studies, neither *O*. *volvulus* mf nor DNA could be detected in the CSF of persons with OAE [17] or in their brains during postmortem studies [8]. However, this could be due to the fact that the study participants had developed their epilepsy many years before, and in the meantime, the parasite might have been eliminated by immune cells of the CNS [2].

## Alternative nodding syndrome hypotheses and research priorities

Several alternative hypotheses have been proposed, but so far, none of them have been confirmed [2]. In postmortem studies, tau deposits were detected in the brain of all persons with NS [18] and in most persons with OAE [8]. Signs of neuroinflammation (gliosis and activated microglia) were noted as well, colocalised with tau-reactive neurofibrillary tangles and threads [8]. In addition, signs of earlier ventriculitis were observed in 8 of 9 persons who died with OAE, suggesting involvement of the choroid plexus as proposed by Duke [12]. Microfilariae in the CSF might gain access to the pituitary gland, where their presence might lead to dwarfism (Nakalanga syndrome) [12]. We hypothesise that the tau deposits are the consequence of a neuroinflammatory reaction induced, directly or indirectly, by *O*. *volvulus*.

**Fig 1:**
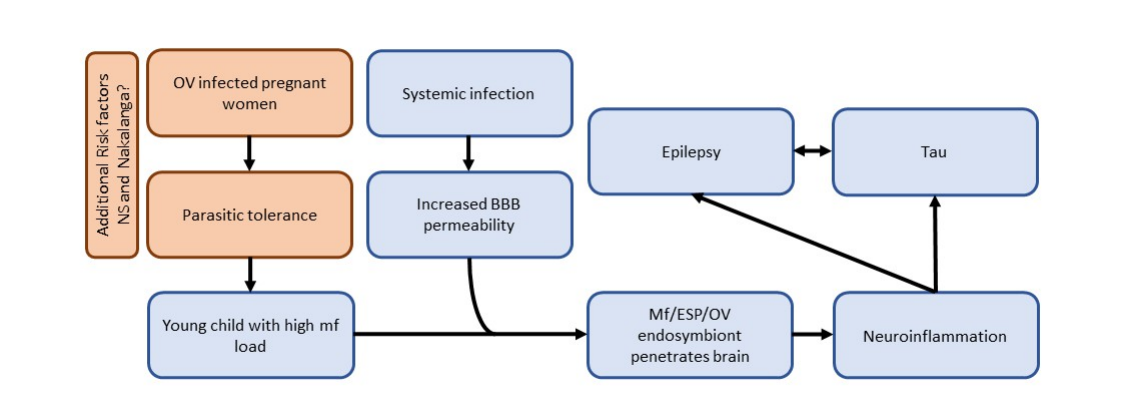
Onchocerciasis hypothesis of OAE, including NS. BBB, blood–brain barrier; ESP, excretory and secretory product; mf, microfilaria; NS, nodding syndrome; OV, *Onchocerca volvulus*.

A systemic infection or physiological stress (e.g., a provoked seizure) in a young child, similar to DEC, may cause CNS inflammation that will increase the permeability of the BBB. In case such children harbour a very high mf load, *O*. *volvulus* mf, secretory/excretory products, or endosymbionts, including viruses, could occasionally cross the weakened BBB causing neuroinflammation, resulting in epilepsy and tau deposits. Thereupon, the epilepsy and tau deposits could sustain each other (Fig 1).

Recently, an additional risk factor for development of NS and Nakalanga was proposed [13]. In a case–control in Uganda, preterm birth was identified as a risk factor for NS [13]. *O*. *volvulus* infection during pregnancy has been associated with an increased risk of spontaneous abortions [13]. Therefore, preterm birth of children who later developed NS may have been the consequence of an *O*. *volvulus* infection in the pregnant mother [2]. Such an *O*. *volvulus* infection during pregnancy may lead to parasite tolerance that can be transmitted in utero [2]. Thereupon, when this child is exposed to *O*. *volvulus* infected blackflies, he/she may develop a very high mf load at a young age, potentially causing NS and/or Nakalanga syndrome, which are the most severe forms of OAE with an earlier epilepsy onset [3].

This hypothesis is currently being investigated, in Cameroon and South Sudan, during a prospective cohort study of children born from *O*. *volvulus* infected and noninfected mothers. These children, not yet eligible for ivermectin treatment, will be followed for a 4-year period and assessed annually for *O*. *volvulus* infection and neurocognitive development. In case of complicated febrile seizures or epilepsy, a lumbar tap will be performed, and collected CSF will be examined for presence of mf, *O*. *volvulus*, and *Wolbachia* DNA.

In addition, we will conduct omics studies to increase our knowledge about the biology of *O*. *volvulus*. With proteomics, we hope to identify *O*. *volvulus* excretory/secretory proteins that could play a role in the pathogenesis of OAE. Moreover, a viral metagenomic study of adult *O*. *volvulus* worms, extracted in Maridi, South Sudan, from nodules from persons with OAE and persons without epilepsy, is planned (ClinicalTrials.gov registration NCT05868551) to identify possible neurotropic viruses. Proteomic and metagenomic studies may not only reveal a potential pathogenetic mechanism of OAE but also lead to new ways to treat and diagnose onchocerciasis.

## Importance to recognise the link between onchocerciasis and epilepsy

Recognition of OAE as a morbidity of onchocerciasis and acceptance that OAE, including NS, can be prevented through strengthening onchocerciasis elimination programs is of paramount importance. The prevention of OAE should be prioritized in public health intervention agendas. Increased awareness about OAE will also improve uptake of CDTi and eventually decrease the burden of onchocerciasis and OAE as well as reducing the time required to eliminate these diseases.
